# Anodal transcranial direct current stimulation of the visual cortex for migraine prevention: a proof-of-concept study

**DOI:** 10.1186/1129-2377-14-S1-P194

**Published:** 2013-02-21

**Authors:** A Viganò, D Magis, SL Sava, V De Pasqua, M Auvé, A Giuliani, A Colosimo, V Di Piero, J Schoenen

**Affiliations:** 1 Sapienza - University of Rome, Italy; 2 ULg - University of Liège, Belgium; 3 Istituto Superiore di Sanità, Italy

## Introduction

Prophylaxis is challenging in migraine because of the low efficiency/tolerability ratio of most drugs [1]. Abnormal excitability of the cerebral cortex seems implicated in migraine pathophysiology [2]. Transcranial direct current stimulation (tDCS) can durably modify the activity of a target cortex and thus be a promising treatment [3]. We have shown that the cerebral cortex, namely the visual cortex, is hyperreactive in migraineurs between attacks and hypothesized that this may be related more to a decreased preactivation level than to hyperexcitability per se [2]. Anodal, rather than cathodal, tDCS might be the stimulation modality of choice in migraine.

## Aims

To explore the effect of anodal tDCS on visual cortex reactivity in healthy volunteers (HV) and migraine patients (EM) and its potentials for migraine prevention.

## Methods

Amplitude and habituation of pattern-reversal visual evoked potentials (VEP) were measured between the 1st and the 6th block of 100 averagings before and after tDCS (1mA; 15 mins) of the visual cortex on HV (n=11) and on EM (n=12) without aura interictally. To study therapeutic potential, we applied tDCS (15 min) on the visual cortex twice/week for 8 weeks in 7 EM with at least 4 attacks/month and a pre-treatment 2 months baseline.

## Results

In HV, tDCS significantly increased the habituation slope of the VEP N1P1 component but had no effect on P1N2. In EM, tDCS tended to increase habituation of both N1P1 and P1N2. At the end of tDCS treatment, there was on average a significant reduction in migraine frequency from 9.14 attacks during the baseline to 5.57 during tDCS (-36.65%, p<0.05). Mean attack duration changed from 124 to 97 min after tDCS (-43.25%, p>0.05).

## Discussion

Anodal tDCS on the visual cortex is thus able to increase habituation of VEP that is reduced in migraineurs interictally. Moreover, 2 weekly sessions of anodal tDCS may have a preventive effect in patients. Hence larger sham-controlled trials with anodal tDCS of the visual cortex are worthwhile in migraine.
